# High-pitch free-breathing triple rule-out CT angiography for acute chest pain: a prospective sequential two-cohort comparison of radiation dose and diagnostic image quality

**DOI:** 10.1186/s13244-026-02390-6

**Published:** 2026-09-04

**Authors:** Juan Yu, Zhoufeng Peng, Xi Yu, Benyang Wang, Wenbing Yang, Wenyang Pan, Yunfei Zha

**Affiliations:** 1 The Department of Radiology, Renmin Hospital of Wuhan University, Wuhan, China; 2 Siemens Healthineers, Shanghai, China

**Keywords:** Chest pain, Computed tomography angiography, Multidetector computed tomography, Radiation dosage

## Abstract

**Objectives:**

To evaluate the feasibility of high-pitch free-breathing triple rule-out CT angiography (TRO-CTA) by comparing image quality and radiation dose with a standard breath-hold protocol in patients with acute chest pain.

**Materials and methods:**

This prospective study enrolled 104 patients with acute chest pain who underwent TRO-CTA on a third-generation dual-source CT scanner, sequentially allocated to high-pitch free-breathing TRO-CTA (pitch = 3.2; Group A, *n* = 52) or standard breath-hold TRO-CTA (standard pitch; Group B, *n* = 52). Objective image quality (attenuation, noise, signal-to-noise ratio [SNR], and contrast-to-noise ratio [CNR]) was assessed across the coronary, pulmonary, and aortic territories. Subjective image quality (3-point scale), effective dose (ED), acquisition time, and clinical diagnoses were compared between groups.

**Results:**

Radiation dose (ED: 1.81 ± 1.33 vs 15.63 ± 6.71 mSv) and acquisition time (0.48 ± 0.05 vs 6.83 ± 1.87 s) were significantly lower in Group A than in Group B (all *p* < 0.001). Vascular attenuation was significantly higher in Group A in the coronary arteries, main pulmonary artery, and ascending aorta (*p* < 0.05), whereas CNR was higher in Group B (all *p* < 0.01). Subjective image quality scores, including coronary diagnostic segment rates (98.0% vs 97.5%), were comparable across all vascular territories (all *p* > 0.05). Clinical diagnoses were similarly distributed between groups (*p* = 0.455).

**Conclusion:**

High-pitch free-breathing TRO-CTA substantially reduces radiation dose while preserving comparable diagnostic image quality, offering a clinically feasible low-dose alternative to standard breath-hold protocols for emergency patients with acute chest pain, particularly those with well-controlled heart rates.

**Key Points:**

**Question**: Conventional TRO-CTA involves high radiation exposure (8.93–17.5 mSv) and breath-holding, posing safety and feasibility challenges in emergency patients with acute chest pain.

**Findings**: The high-pitch free-breathing protocol reduced effective dose by 88% and acquisition time from 6.83 to 0.48 s while providing comparable diagnostic image quality.

**Critical relevance statement**: High-pitch free-breathing TRO-CTA may offer a low-dose imaging option on dual-source CT, with the potential to support more time-efficient emergency evaluation of acute chest pain.

**Graphical Abstract:**

**Figure:**
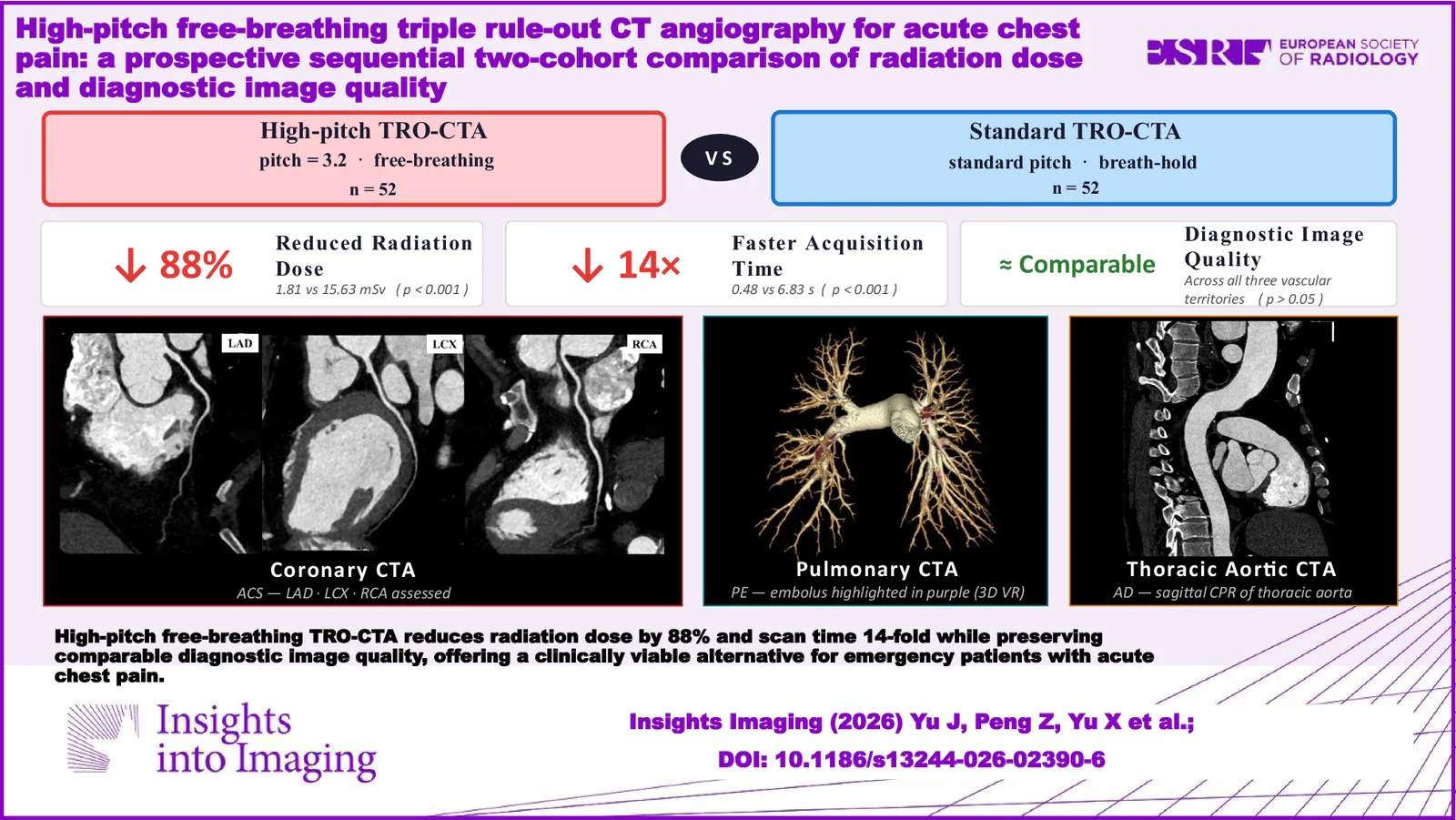

## Introduction

Acute chest pain is a common and critical presentation in emergency departments, necessitating rapid diagnostic evaluation due to its potential for life-threatening complications [1]. The primary differential diagnoses include acute coronary syndrome (ACS), pulmonary embolism (PE), and aortic dissection (AD) [2]. Prompt identification of the underlying etiology and risk stratification are crucial for reducing morbidity and mortality [3].

Triple rule-out CT angiography (TRO-CTA) has emerged as a comprehensive noninvasive imaging modality for the simultaneous assessment of ACS, PE, and AD in patients with acute chest pain [4, 5]. However, conventional TRO-CTA protocols are associated with relatively high radiation exposure, with effective doses typically ranging from 8.93 to 17.5 mSv [6, 7]. Given the increased lifetime cancer risk associated with ionizing radiation, there is a growing imperative to optimize CT protocols to minimize radiation dose while maintaining diagnostic image quality [8]. Moreover, conventional protocols require prolonged breath-holding, which poses additional challenges for dyspneic patients in the emergency setting.

To address this challenge, various dose-reduction strategies have been implemented, including low-kilovoltage (kV) settings, ECG-gated tube current modulation, optimization of the ECG-pulsing window, and advanced iterative reconstruction algorithms [9–11]. Beyond these parameter-level optimizations, the advent of dual-source CT technology has enabled a high-pitch dual-spiral acquisition technique that allows rapid acquisition of an ECG-gated dataset of the entire chest in less than one second with significantly reduced radiation exposure [12]. Although this high-pitch technique is critical for coronary CT angiography, planning of transcatheter aortic valve implantation, CT pulmonary angiography, and aortic CT angiography, its application to TRO-CTA remains limited, constrained by small sample sizes and the absence of direct comparison with standard TRO-CTA protocols [13–18].

Therefore, this prospective study aimed to evaluate the feasibility of high-pitch free-breathing TRO-CTA by comparing image quality and radiation dose with standard breath-hold TRO-CTA in patients with acute chest pain.

## Materials and methods

### Patient population

This study was conducted in accordance with the Declaration of Helsinki and approved by the Ethics Committee of Renmin Hospital of Wuhan University (No. WDRY2024-K182). Written informed consent was obtained from all participants before their enrollment.

**Fig. 1:**
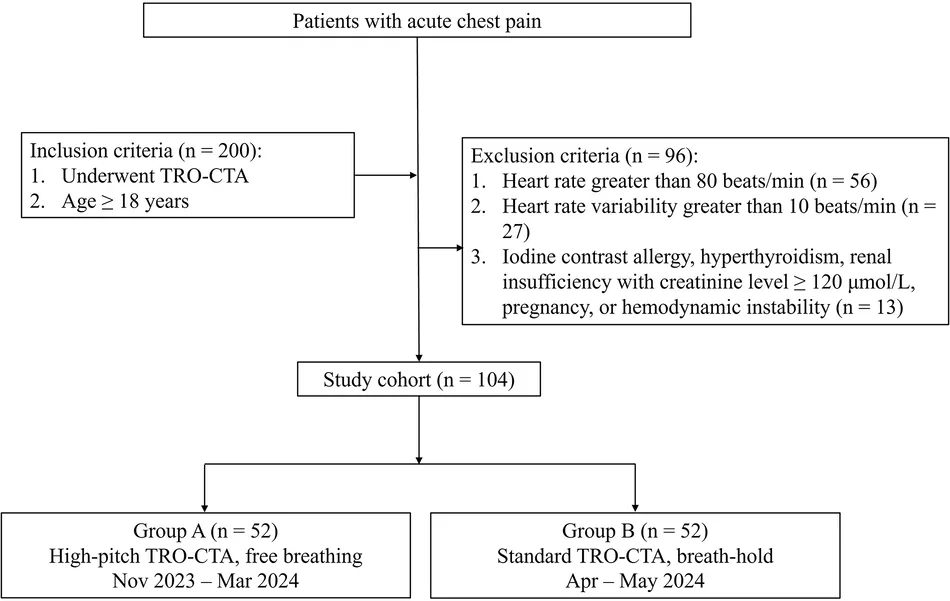
Flowchart of the study population

A total of 104 consecutive patients (53 women, 51 men; mean age, 60 ± 12 years) undergoing TRO-CTA were prospectively enrolled between November 2023 and May 2024. Inclusion criteria were age ≥ 18 years and clinical suspicion of acute thoracic vascular disease (ACS, PE, or AD). Heart rate (HR) and heart rate variability (HRV) were recorded before scanning. HRV was defined as the difference between the minimum and maximum HR during the 10 beats preceding the scan. Exclusion criteria were as follows: (i) HR > 80 beats per minute (bpm) (*n* = 56); (ii) HRV > 10 bpm (*n* = 27); (iii) iodine contrast allergy (*n* = 3); (iv) hyperthyroidism (*n* = 2); (v) renal insufficiency (creatinine ≥ 120 μmol/L) (*n* = 4); (vi) pregnancy (*n* = 1); (vii) hemodynamic instability (*n* = 3). Patients were sequentially allocated to two groups according to enrollment period. Group A (*n* = 52; November 2023–March 2024) underwent high-pitch TRO-CTA with free breathing (pitch = 3.2); Group B (*n* = 52; April–May 2024) was examined using standard TRO-CTA with breath-hold (Fig. 1). No additional HR–lowering or vasodilator medications were administered beyond routine treatment.

### Acquisition protocol

All CT examinations were performed on a third-generation dual-source CT scanner (SOMATOM Force; Siemens Healthineers, Forchheim, Germany) in the supine position with feet-first orientation. The scan parameters were as follows: detector collimation of 192 × 0.6 mm; gantry rotation time of 0.25 s; automated tube current modulation (CARE Dose4D); and automated tube voltage selection (CARE kV). Group A underwent prospectively ECG-triggered high-pitch spiral acquisition (pitch = 3.2) at 65% of the R-R interval during free breathing. Group B underwent retrospective ECG-gated scanning at standard pitch with an acquisition window of 30%–75% of the R-R interval during breath-hold. Scan coverage extended from the lung apices to the costophrenic recesses in both groups.

A three-phase contrast injection protocol was employed: a 50-mL bolus of iopromide (Ultravist 370 mg I/mL; Bayer Schering Pharma, Berlin, Germany) at 4.5 mL/s, followed by 25 mL at 3 mL/s and a 30-mL saline flush at the same rate. In Group A, the second-phase contrast volume was reduced to 20 mL owing to the shorter acquisition time. Bolus tracking was performed with the region of interest (ROI) placed in the ascending aorta, using a trigger threshold of 100 Hounsfield units (HU) and a scan delay of 4–5 s in both groups. All datasets were reconstructed using Advanced Modeled Iterative Reconstruction (ADMIRE, strength level 3) with a slice thickness and increment of 0.75 mm. The Bv36 vascular kernel was applied for coronary artery (CA) evaluation, and the Bv40 kernel for assessment of the aorta and pulmonary arteries. Acquisition time, HR, CT volume dose index (CTDI_vol_), and dose-length product (DLP) were recorded from the scanner console for each patient. Effective dose (ED) was calculated by multiplying DLP by a chest-specific conversion factor (*k* = 0.0188 mSv·mGy⁻¹·cm⁻¹ for Group A and 0.0180 mSv·mGy⁻¹·cm⁻¹ for Group B), as reported by Sommer et al [12].

### Image quality

#### Objective image quality

Image quality was independently assessed by two radiologists, each with 6 years of experience in cardiovascular CTA, who were blinded to the scanning protocols. Multiplanar reconstruction and quantitative analysis were performed using Syngo.via (VB40A; Siemens Healthineers, Erlangen, Germany). Attenuation and standard deviation (SD) were measured using circular ROIs at 14 vascular locations, including the left main coronary artery (CA-LM), left anterior descending artery (CA-LAD), left circumflex artery (CA-LCX), right coronary artery (CA-RCA), main pulmonary artery (PA-MA), left and right main pulmonary arteries (PA-LPA, PA-RPA), five lobar pulmonary arteries (left upper [PA-LULA], left lower [PA-LLLA], right upper [PA-RULA], right middle [PA-RMLA], and right lower [PA-RLLA]), ascending aorta (TA-AA), descending aorta (TA-DA), as well as subcutaneous fat and paravertebral muscle at the level of the aortic root. ROIs were placed at the mid-segment of each coronary and PA branch, with care taken to avoid plaques, stenosis, or adjacent lesions. For the ascending and descending aorta, measurements were obtained approximately 1 cm distal to the tracheal bifurcation. The size of ROIs was adjusted to match the arterial lumen diameter to minimize artifacts from adjacent structures. The SD of subcutaneous fat was used as background noise for CA measurements, whereas the SD of paravertebral muscle was used for PA and aortic measurements. The mean of the two readers’ measurements was used for subsequent analysis. Signal-to-noise ratio (SNR) and contrast-to-noise ratio (CNR) were calculated as follows:

$${SN}{R}_{{ROI}}=\frac{C{T}_{{ROI}}}{S{D}_{{background}}}$$

$${CNR}_{ROI}=\frac{{CT}_{ROI}-{{CT}_{background}}}{{{SD}}_{{background}}}$$

#### Subjective image quality

CAs were analyzed according to the 18-segment model proposed by the Society of Cardiovascular Computed Tomography [19]. Image quality of CAs, PAs, and TAs was independently assessed by two radiologists using a 3-point semi-quantitative scale based on overall image clarity and motion artifacts: 3, excellent image quality without motion artifacts; 2, diagnostically acceptable image quality with mild vessel wall blurring; and 1, non-diagnostic image quality with severe vessel wall blurring or double contours precluding diagnostic interpretation. The lower score assigned by the two readers was recorded for analysis.

### Statistical analysis

Statistical analyses were performed using SPSS (version 22.0, IBM, Chicago, IL, USA) with two-tailed *p* < 0.05 considered statistically significant. Normality was assessed using the Shapiro–Wilk test. Continuous variables were reported as mean ± SD when normally distributed, or median (interquartile range) otherwise; categorical variables were expressed as frequencies and compared using the chi-square test, or Fisher’s exact test when expected cell frequencies were less than 5. The Mann–Whitney *U*test was used for qualitative image quality scores; the unpaired *t*-test for continuous variables (age, BMI, HR, acquisition time, HU values, SNR, CNR, and radiation dose metrics: CTDI_vol_, DLP, ED). Inter-observer agreement was assessed using kappa analysis for subjective image quality scores (> 0.80, excellent; 0.61–0.80, good; 0.41–0.60, moderate; 0.21–0.40, fair; ≤ 0.20, poor) and Bland–Altman analysis for objective measurements (SNR and CNR), with mean difference and 95% limits of agreement reported.

## Results

### Study population

A total of 104 patients were analyzed, including 52 patients in each group (Fig. 1). Patient characteristics are summarized in Table 1. There were no significant differences in age, sex, BMI, or mean HR between the groups. Mean acquisition time was significantly shorter in Group A (0.48 ± 0.05 s) compared with Group B (6.83 ± 1.87 s, *p* < 0.001).

**Table 1:** Patient characteristics

Parameter	Group A	Group B	*p* value
Total	52	52	
Age, years	60.0 ± 12.25	60.6 ± 12.00	0.815
Sex			0.556
Male	27	24	
Female	25	28	
BMI, kg/m^2^	25.34 ± 3.11	25.43 ± 2.58	0.160
HR, bpm	66.75 ± 6.82	68.02 ± 9.17	0.425
HR distribution			0.598
< 60 bpm	8	6	
60–69 bpm	23	20	
≥ 70 bpm	21	26	
Acquisition time, s	0.48 ± 0.05	6.83 ± 1.87	< 0.001

*BMI* body mass index, *HR* heart rate, *bpm* beats per minute

Data are mean ± SD

*p*-values were determined by the independent *t*-test for continuous variables and the chi-square test for categorical variables

### Objective image quality

**Fig. 2:**
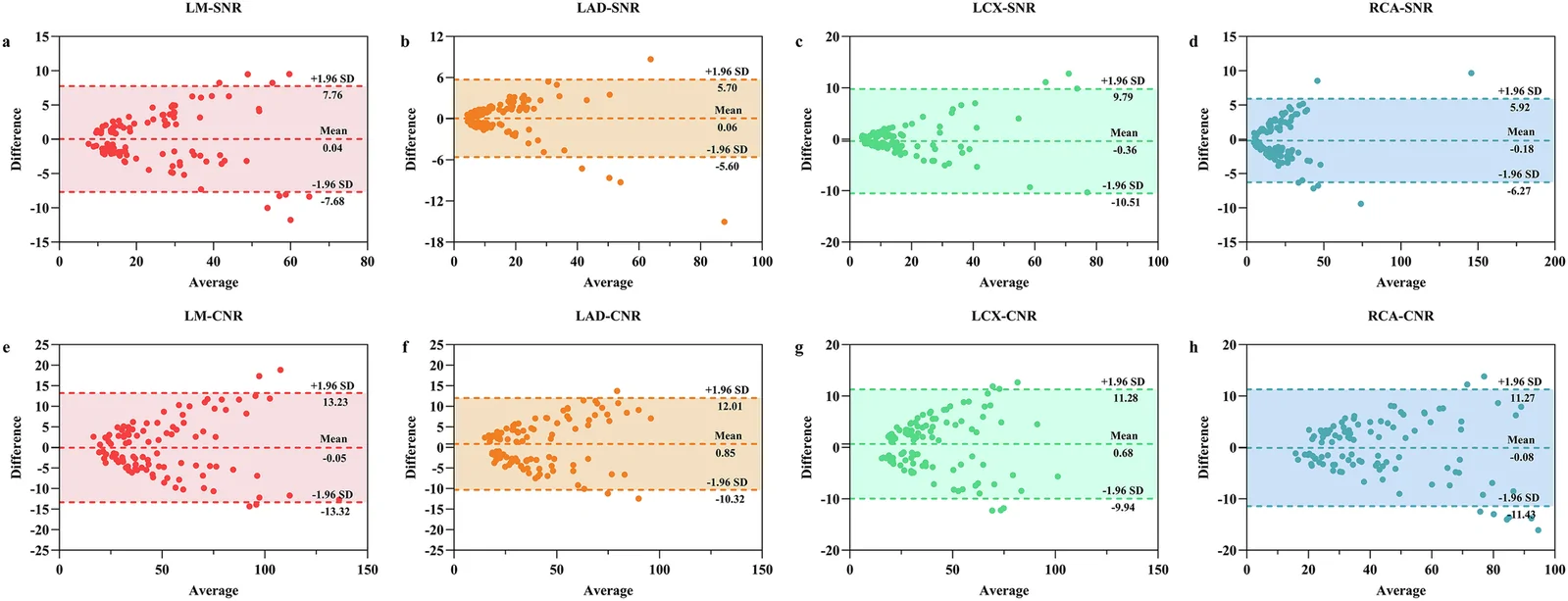
Bland–Altman plot for assessing inter-observer agreement between radiologist A and radiologist B in measuring SNR (**a**–**d**) and CNR (**e**–**h**) of coronary arteries. The *X*-axis is the mean of the two radiologists’ measurements. The *Y*-axis is the difference between measurements. The central dashed line indicates the mean difference. The upper and lower dashed lines represent the 95% limits of agreement

**Fig. 3:**
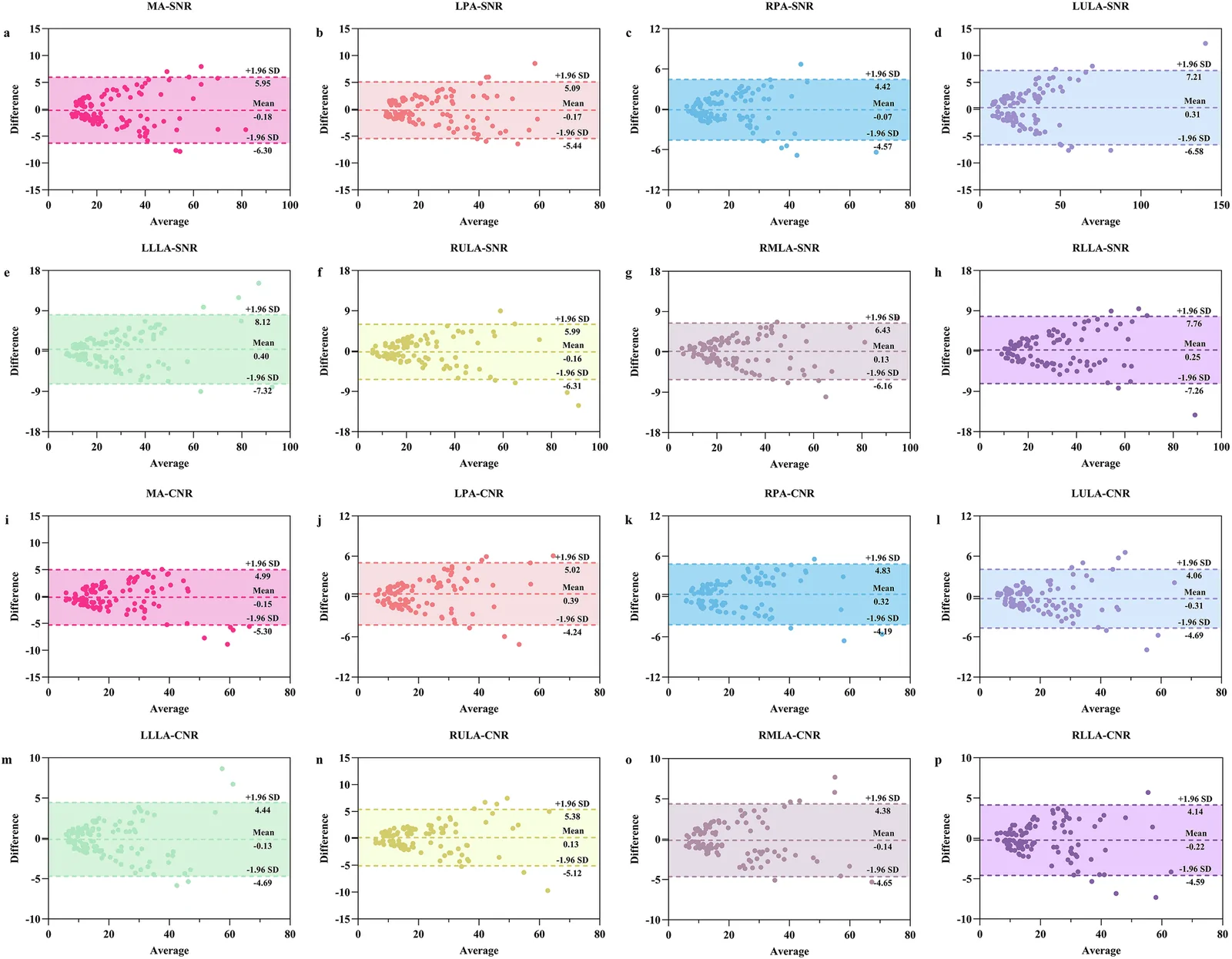
Bland–Altman plot for assessing inter-observer agreement between radiologist A and radiologist B in measuring SNR (**a**–**h**) and CNR (**i**–**p**) of pulmonary arteries. The *X*-axis is the mean of the two radiologists’ measurements. The *Y*-axis is the difference between measurements. The central dashed line indicates the mean difference. The upper and lower dashed lines represent the 95% limits of agreement

**Fig. 4:**
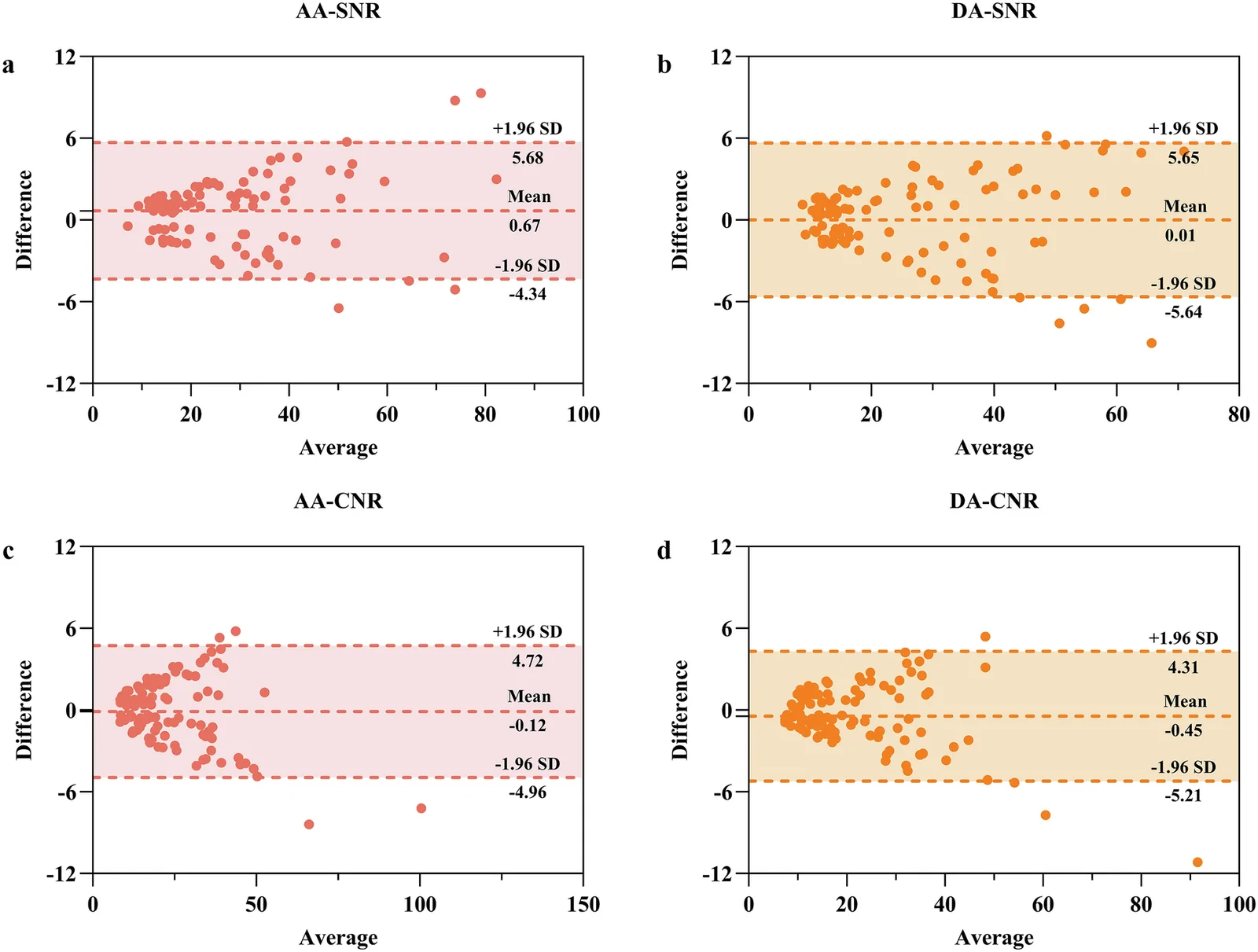
Bland–Altman plot for assessing inter-observer agreement between radiologist A and radiologist B in measuring SNR (**a**, **b**) and CNR (**c**, **d**) of thoracic aorta (TA). The *X*-axis is the mean of the two radiologists’ measurements. The *Y*-axis is the difference between measurements. The central dashed line indicates the mean difference. The upper and lower dashed lines represent the 95% limits of agreement

Quantitative analysis revealed significantly higher mean attenuation values in Group A in the coronary arteries (LM, LAD, LCX, and RCA), PA-MA, and TA-AA (all *p* < 0.05). The remaining vascular segments showed a similar trend toward higher attenuation in Group A without reaching statistical significance. Noise was higher in Group A in all vascular segments except CA-LAD (all *p* < 0.05; Table 2). Group B showed higher SNR in all segments except CA-LAD, CA-LCX, and CA-RCA (all *p* < 0.05), and higher CNR across all vascular territories (all *p* < 0.01). The minimum CNR was 5.65 in Group A (Table S1). Bland–Altman analysis demonstrated inter-observer agreement for SNR and CNR measurements, with mean biases ranging from −0.36 to 0.67 and −0.45 to 0.85, respectively. The 95% limits of agreement ranged from −10.51 to 9.79 for SNR and from −13.32 to 13.23 for CNR (Figs. 2–4).

**Table 2:** Mean attenuation and noise

Criteria	Group A	Group B	*p* value
Mean attenuation			
CA-LM	528.25 ± 103.23	490.52 ± 81.50	0.041
CA-LAD	417.08 ± 89.80	382.00 ± 56.88	0.019
CA-LCX	425.61 ± 85.89	373.61 ± 50.32	< 0.001
CA-RCA	469.19 ± 94.90	405.21 ± 78.06	< 0.001
PA-MA	526.21 ± 125.12	466.81 ± 133.73	0.021
PA-LPA	491.15 ± 111.49	445.63 ± 130.74	0.059
PA-RPA	503.71 ± 120.10	455.54 ± 135.13	0.057
PA-LULA	485.80 ± 119.07	436.23 ± 135.17	0.050
PA-LLLA	473.73 ± 115.36	428.84 ± 131.86	0.068
PA-RULA	493.76 ± 123.15	458.42 ± 138.34	0.172
PA-RMLA	475.75 ± 117.83	448.71 ± 149.33	0.308
PA-RLLA	480.71 ± 119.06	440.60 ± 138.22	0.116
TA-AA	544.06 ± 106.75	502.44 ± 84.42	0.030
TA-DA	497.96 ± 93.79	471.15 ± 81.37	0.123
Noise			
CA-LM	38.94 ± 8.29	14.81 ± 4.84	< 0.001
CA-LAD	42.11 ± 23.85	33.79 ± 20.83	0.061
CA-LCX	42.23 ± 25.47	29.40 ± 18.97	0.004
CA-RCA	37.44 ± 28.38	21.33 ± 12.44	< 0.001
PA-MA	33.81 ± 7.60	12.23 ± 4.28	< 0.001
PA-LPA	34.87 ± 7.21	13.96 ± 5.02	< 0.001
PA-RPA	43.08 ± 13.46	19.19 ± 9.60	< 0.001
PA-LULA	27.92 ± 11.01	13.92 ± 7.96	< 0.001
PA-LLLA	32.17 ± 10.88	11.62 ± 4.71	< 0.001
PA-RULA	32.96 ± 16.16	16.66 ± 9.64	< 0.001
PA-RMLA	28.33 ± 10.08	14.81 ± 12.23	< 0.001
PA-RLLA	28.90 ± 8.83	11.11 ± 4.63	< 0.001
TA-AA	35.96 ± 10.01	14.02 ± 4.75	< 0.001
TA-DA	36.54 ± 6.40	13.19 ± 4.41	< 0.001

*CA-LM* left main coronary artery, *CA-LAD* left anterior descending artery, *CA-LCX* left circumflex artery, *CA-RCA* right coronary artery, *PA-MA* main pulmonary artery, *PA-LPA* left main pulmonary artery, *PA-RPA* right main pulmonary artery, *PA-LULA* left upper lobar artery, *PA-LLLA* left lower lobar artery, *PA-RULA* right upper lobar artery, *PA-RLLA* right lower lobar artery, *PA-RMLA* right middle lobar artery, *TA-AA* ascending aorta, *TA-DA* descending aorta

### Subjective image quality

In Group A, 699 of 713 (98.0%) CA segments were rated as diagnostic; in Group B, 705 of 723 (97.5%) were diagnostic (Table 3). Ten patients (19.2%) in Group A had at least one non-diagnostic segment, compared with 7 patients (13.5%) in Group B (*p* > 0.05). Among patients with non-diagnostic segments, 8/10 (80%) in Group A and 4/7 (57.1%) in Group B had an HR > 70 bpm during scanning. Image quality ratings for PAs and TAs were ≥ 2 in both groups. Minor motion artifacts were identified in 4 patients (7.7%) in Group A and 5 patients (9.6%) in Group B for the TAs, and in 4 patients in each group for PAs. No statistically significant differences were observed between groups (Table 4). Overall inter-observer agreement was excellent (κ = 0.896).

**Table 3:** Comparison of subjective CA scores between high-pitch TRO-CTA and standard TRO-CTA

	Group A (3/2/1)	*k*	Group B (3/2/1)	*k*	*p* value
RCA-1	39/12/1 (*n* = 52)	0.892^#^	38/11/3 (*n* = 52)	0.919^#^	0.743
RCA-2	28/18/6 (*n* = 52)	0.802^#^	21/27/4 (*n* = 52)	0.961^#^	0.323
RCA-3	37/15/0 (*n* = 52)	0.952^#^	38/13/1 (*n* = 52)	0.843^#^	0.877
RCA-4	34/13/1 (*n* = 48)	0.969^#^	39/8/1 (*n* = 48)	0.899^#^	0.246
RCA-16	31/11/0 (*n* = 42)	0.970^#^	30/6/2 (*n* = 38)	0.966^#^	0.696
LM-5	49/3/0 (*n* = 52)	0.790^*^	49/2/1 (*n* = 52)	0.790^*^	0.981
LAD-6	49/3/0 (*n* = 52)	0.790^*^	49/2/1 (*n* = 52)	0.790^*^	0.981
LAD-7	51/1/0 (*n* = 52)	1.000^#^	47/3/2 (*n* = 52)	0.812^#^	0.099
LAD-8	48/4/0 (*n* = 52)	0.847^#^	48/4/0 (*n* = 52)	0.768^*^	1.000
LAD-9	44/6/0 (*n* = 50)	0.949^#^	38/12/1 (*n* = 51)	0.894^#^	0.079
LAD-10	31/4/0 (*n* = 35)	0.985^#^	28/7/0 (*n* = 35)	1.000^#^	0.328
LCX-11	46/5/1 (*n* = 52)	1.000^#^	48/3/1 (*n* = 52)	0.821^#^	0.516
LCX-12	26/16/3 (*n* = 45)	0.970^#^	27/21/0 (*n* = 48)	0.966^#^	0.894
LCX-13	44/6/2 (*n* = 52)	0.835^#^	43/8/1 (*n* = 52)	0.917^#^	0.832
LCX-14	8/1/0 (*n* = 9)	1.000^#^	16/4/0 (*n* = 20)	1.000^#^	0.565
LCX-15	4/0/0 (*n* = 4)	1.000^#^	3/0/0 (*n* = 3)	1.000^#^	1.000
LCX-18	2/0/0 (*n* = 2)	1.000^#^	–(*n* = 0)	–	
RI-17	10/0/0 (*n* = 10)	1.000^#^	10/2/0 (*n* = 12)	0.814^#^	0.186
Total	581/118/14 (*n* = 713)	0.892^#^	572/133/18 (*n* = 723)	0.886^#^	0.152

*RCA* right coronary artery, *LM* left main coronary artery, *LAD* left anterior descending artery, *LCX* left circumflex artery, *RI* ramus intermedius artery

Coronary segments are numbered according to the 18-segment model of the Society of Cardiovascular Computed Tomography

Subjective image quality was evaluated using a 3-point semi-quantitative scale: 3 = excellent (no motion artifacts); 2 = diagnostically acceptable (mild vessel wall blurring); and 1 = non-diagnostic (severe vessel wall blurring or double contours precluding diagnostic interpretation)

^#^ Excellent agreement; ^*^ good agreement

**Table 4:** Comparison of subjective PA and TA scores between high-pitch TRO-CTA and standard TRO-CTA

Quality rating score	PA (Group A)	PA (Group B)	*p* value	TA (Group A)	TA (Group B)	*p* value
3	48	48	1.000	48	47	0.729
2	4	4		4	5	
1	0	0		0	0	
Total	52	52		52	52	

*PA* pulmonary artery, *TA* thoracic aorta

Subjective image quality was evaluated using a 3-point semi-quantitative scale: 3 = excellent (no motion artifacts); 2 = diagnostically acceptable (mild vessel wall blurring); and 1 = non-diagnostic (severe vessel wall blurring or double contours precluding diagnostic interpretation)

### Clinical diagnoses

The clinical diagnoses identified by TRO-CTA were comparable between the two groups (Table S2). Obstructive coronary artery disease (CAD) was identified in 4 patients (7.7%) in Group A and 6 patients (11.5%) in Group B. Acute aortic syndrome (AAS, including aortic intramural hematoma and penetrating aortic ulcer) was detected in 4 patients (7.7%) in each group, and PE in 1 patient (1.9%) in Group A and 4 patients (7.7%) in Group B. Overall, any TRO acute finding (obstructive CAD, PE, or AAS) was present in 8 patients (15.4%) in Group A and 12 patients (23.1%) in Group B (*p* = 0.455). The remaining patients had either non-obstructive incidental findings or unremarkable imaging findings. No patient in either group required additional imaging due to non-diagnostic image quality.

### Radiation exposure

**Fig. 5:**
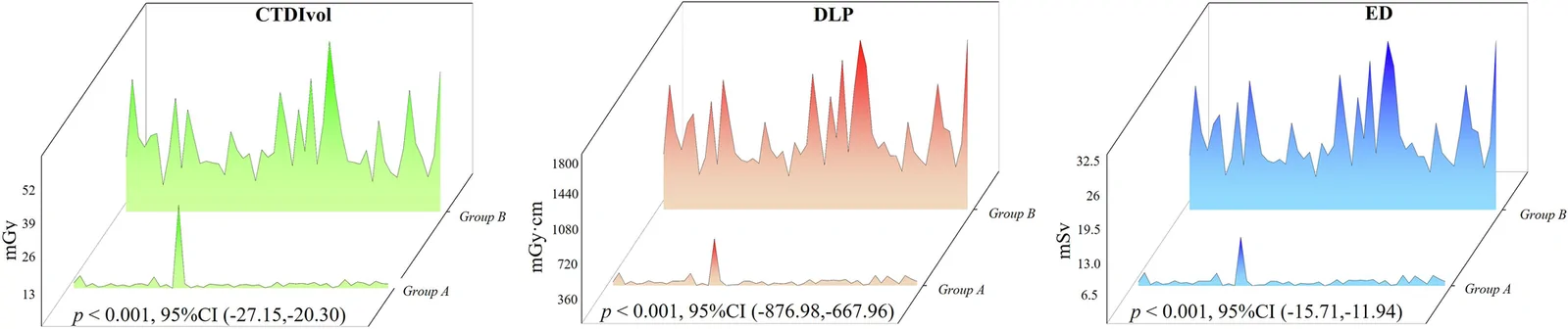
Comparison of radiation exposure parameters between high-pitch (Group A) and standard (Group B) TRO-CTA protocols

Radiation exposure was significantly lower in Group A compared with Group B across all dose parameters (all *p* < 0.001). The mean ED was 1.81 ± 1.33 mSv in Group A vs 15.63 ± 6.71 mSv in Group B, representing an 88% reduction. Detailed dose parameters (CTDI_vol_, DLP, and ED) are presented in Table 5 and Fig. 5.

**Table 5:** Comparison of radiation exposure

Parameter	Group A	Group B	Mean Difference (95% CI)	*p* value
CTDI_vol_, mGy	3.27 ± 4.13	26.99 ± 11.75	−23.72 (−27.15 to −20.30)	< 0.001
DLP, mGy·cm	96.05 ± 70.79	868.51 ± 373.29	−772.46 (−876.98 to −667.96)	< 0.001
ED, mSv	1.81 ± 1.33	15.63 ± 6.71	−13.82 (−15.71 to −11.94)	< 0.001

*CTDIvol* volume CT dose index, *DLP* dose-length product, *ED* effective dose, *CI* confidence interval

## Discussion

High-pitch helical scanning (pitch = 3.2) on a third-generation dual-source CT scanner reduced radiation dose by 88% and acquisition time to 0.48 s compared with standard helical scanning. This allowed free-breathing acquisition while maintaining comparable diagnostic image quality in patients with acute chest pain. These findings may help address the key limitations of conventional TRO-CTA in emergency settings, including high radiation exposure and breath-holding requirements. Uniform vascular enhancement across coronary, aortic, and pulmonary circulations is crucial for diagnostic TRO-CTA image quality [20]. Prior studies have established minimum attenuation thresholds of > 200 HU for the reliable diagnosis of AD and > 250 HU for PE and coronary atherosclerotic lesions [21, 22]. Our results indicated that objective assessment presented a slight reduction in image quality with high-pitch TRO-CTA compared with the standard protocol, although no significant difference in subjective image quality was observed. All measured vascular segments showed CT attenuation values above 250 HU, exceeding these diagnostic standards. Although the PAs showed lower CNR values than other territories, the minimum CNR of 5.65 remained above the threshold of 5.0 previously reported by Holmquist et al as sufficient for reliable PE detection [23]. Importantly, Bland–Altman analysis demonstrated good inter-observer agreement (Figs. 2–4), supporting the reliability of the measurements. Therefore, the high-pitch scanning mode on third-generation dual-source CT provides diagnostically adequate image quality for TRO-CTA despite slightly increased noise.

**Fig. 6:**
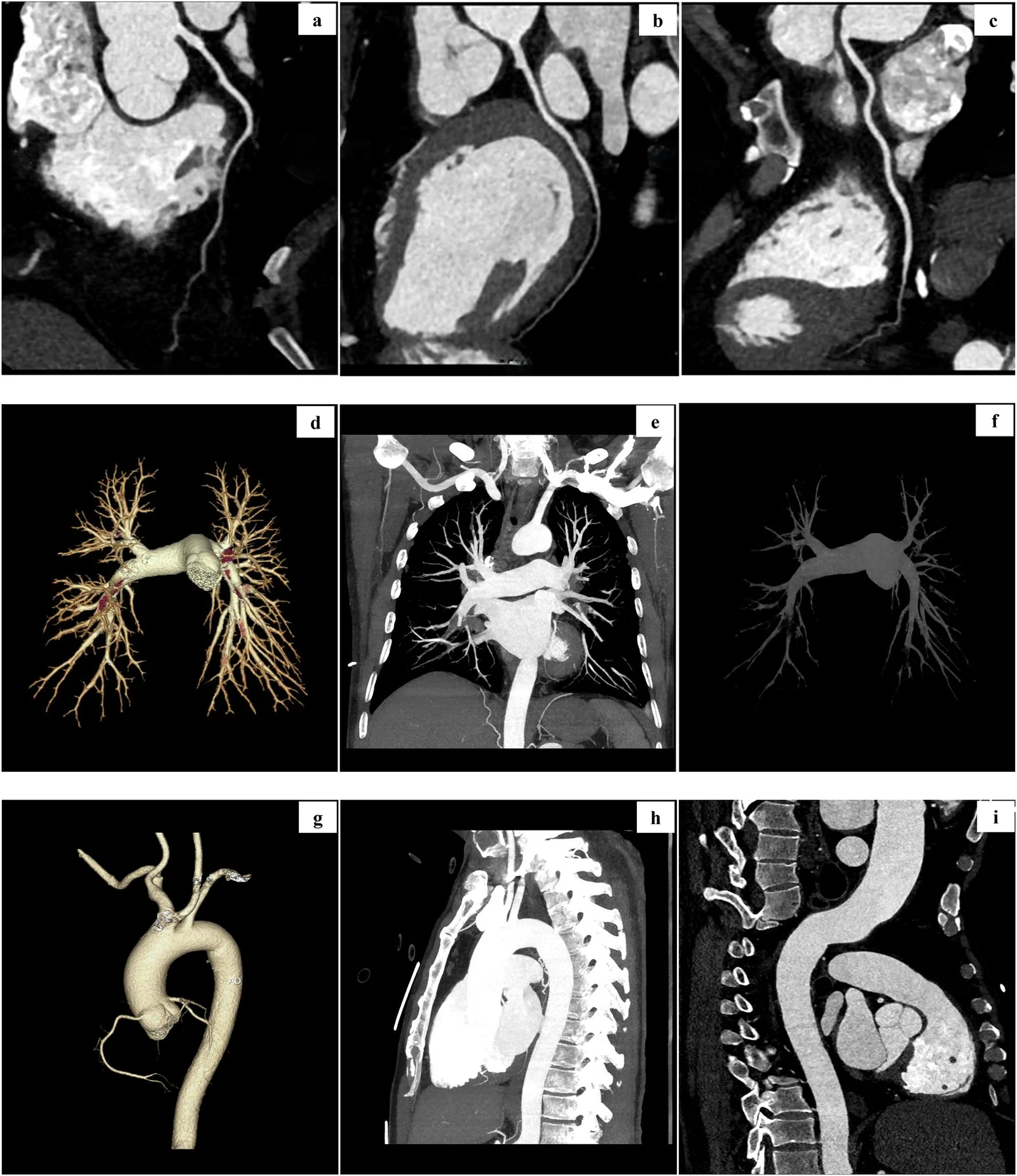
A patient who presented with acute chest pain and dyspnea underwent TRO-CTA using a high-pitch protocol on a third-generation dual-source CT system. **a**–**c** Curved planar reconstruction of coronary arteries showing no plaques or stenosis in the left anterior descending artery (**a**), the left circumflex artery (**b**), and the right CA (**c**). **d**–**f** PE in the segmental and subsegmental arteries of both lungs. Three-dimensional volume rendering (**d**) with emboli shown in purple, coronal maximum intensity projection (**e**), and pulmonary arterial maximum intensity projection (**f**). **g**–**i** No motion artifacts or indications of dissection in the ascending or descending TA

The diagnostic performance for coronary segments was high and comparable between groups (98.0% vs 97.5%, *p* > 0.05). The slightly higher rate observed in the high-pitch protocol compared with previous reports may be attributed to the improved temporal resolution of third-generation dual-source CT [16, 18]. Furthermore, we found that the majority of non-diagnostic segments occurred in patients with HRs exceeding 70 bpm. The clinical utility of the high-pitch mode is limited in such patients because it depends on an adequate diastolic phase for image acquisition. This phase becomes progressively shorter at higher HRs, resulting in motion artifacts and reduced image quality [12]. Consistent with this mechanism, HRs > 70 bpm were more prevalent among patients with non-diagnostic segments in Group A than in Group B (80.0% vs 57.1%). Nevertheless, the overall low non-diagnostic rate underscores the capability of modern CT hardware to largely compensate for this limitation. In contrast, the image quality of PAs and TAs was diagnostically sufficient in all cases for both groups, owing to their relative insensitivity to motion. As previously reported [14, 24], no respiratory motion artifacts compromising the diagnostic evaluation were observed in the PAs and TAs of Group A. Therefore, the high-pitch protocol can be considered a clinically feasible alternative to the standard protocol for TRO-CTA, particularly in patients with well-controlled HRs (Fig. 6).

Radiation exposure remains a significant concern in TRO-CTA. While reducing tube voltage (e.g., to 100 kV) effectively lowers dose [25], it may concurrently increase image noise. Alternative techniques, such as the all-axial scan mode, have been reported to achieve a radiation dose of approximately 2.67 mSv [26]. In this study, the high-pitch protocol achieved a markedly lower dose of 1.81 ± 1.33 mSv, comparable to that of non-contrast chest CT [12]. This effect may be attributable to the combination of prospective ECG triggering and high-pitch acquisition, which shortens acquisition time and thereby reduces X-ray exposure. Accordingly, the high-pitch technique may represent a feasible strategy for dose reduction in TRO-CTA.

Beyond radiation exposure, the volume of iodinated contrast medium represents another critical safety consideration in TRO-CTA. While essential for diagnostic efficacy [27], its association with adverse effects such as contrast-induced nephropathy (CIN) has spurred efforts toward contrast volume reduction [28]. Early protocols used up to 120 mL [25], whereas Chen et al [26] and Wang et al [7] reported feasibility with less than 80 mL—which is still above our 70 mL protocol. Notably, our results revealed that using a lower injection volume in combination with a high-pitch scanning mode yielded higher vascular attenuation compared with the standard-pitch protocol (75 mL). This may be partly explained by the ultrashort acquisition time, which minimizes temporal variation in contrast concentration and enables imaging of all vascular territories at near-peak enhancement. Our findings suggest that the high-pitch technique may permit further optimization of contrast protocols in TRO-CTA.

The recent introduction of photon-counting detector CT (PCD-CT) marks a major step forward in cardiovascular CT, offering improved spatial resolution and dose efficiency compared with energy-integrating detector (EID) CT. High-pitch PCD-CT has demonstrated substantial reductions in radiation dose and contrast volume across coronary, pulmonary, and aortic CT angiography [29–32]. However, these investigations have predominantly addressed single-territory examinations on PCD-CT systems that remain available in only a limited number of centers, leaving TRO-CTA, which uniquely requires concurrent optimization of all three vascular territories, underexplored. Against this background, our study demonstrates that substantial dose reduction in TRO-CTA can also be achieved through protocol optimization on widely available CT scanners, providing a clinically feasible strategy. Future studies extending the high-pitch free-breathing strategy to PCD-CT for TRO-CTA may further refine low-dose comprehensive cardiovascular imaging in acute chest pain.

This study has some limitations. First, the two cohorts were sequentially allocated by enrollment period. While both groups were consecutive, recruited under the same eligibility criteria, and showed no significant baseline differences, this non-randomized design cannot fully exclude unmeasured confounders. Second, although final clinical diagnoses are reported and comparably distributed between cohorts, this study was not designed to evaluate diagnostic accuracy against an invasive reference standard. Validation in future studies with reference standards and clinical outcome data would further strengthen the clinical applicability of this protocol. Third, because the high-pitch acquisition depends on a sufficient diastolic window, the protocol applies only to patients with stable hemodynamics and well-controlled HRs, which may limit its applicability to broader emergency populations in whom sympathetic-driven tachycardia is common. Fourth, the contrast chase volume in Group A was reduced from 25 mL to 20 mL to accommodate the substantially shorter acquisition time. The higher attenuation observed in Group A may be partially influenced by this optimized bolus geometry rather than solely being driven by the high-pitch acquisition itself. Although this adaptation represents a practical clinical workflow for ultra-fast scanning, future studies with precisely matched injection protocols are warranted to isolate the standalone contribution of the high-pitch technique. Finally, this single-center study was conducted exclusively on a third-generation dual-source CT scanner from a single vendor (SOMATOM Force, Siemens Healthineers), which features specific ultra-high-pitch capabilities. Whether similar results can be achieved on CT platforms from other vendors with comparable high-pitch capabilities warrants further investigation.

## Conclusion

In summary, TRO-CTA using the high-pitch protocol with a third-generation dual-source CT system provides diagnostically adequate image quality in free-breathing patients while substantially reducing both radiation exposure and acquisition time. These findings support the high-pitch acquisition technique as a clinically feasible alternative to standard breath-hold TRO-CTA for emergency patients with acute chest pain, particularly for those with well-controlled HRs.

## Supplementary information

ELECTRONIC SUPPLEMENTARY MATERIAL

## Data Availability

The datasets used and/or analyzed during the current study are available from the corresponding author on reasonable request.

## Abbreviations

bpm: Beats per minute
CA: Coronary artery
CNR: Contrast-to-noise ratio
CTA: Computed tomography angiography
CTDIvol: Computed tomography volume dose index
DLP: Dose-length product
HR: Heart rate
HU: Hounsfield unit
kV: Kilovoltage
PA: Pulmonary artery
PE: Pulmonary embolism
ROI: Region of interest
SD: Standard deviation
SNR: Signal-to-noise ratio
TA: Thoracic aorta
TRO-CTA: Triple rule-out computed tomography angiography

## References

[1] Sarto G, Simeone B, Spadafora L et al (2024) Management of acute chest pain in the Emergency Department: benefits of coronary computed tomography angiography. Int J Cardiovasc Imaging 40:2447–2457 39541059 10.1007/s10554-024-03274-w

[2] Gulati M, Levy PD, Mukherjee D et al (2021) 2021 AHA/ACC/ASE/CHEST/SAEM/SCCT/SCMR guideline for the evaluation and diagnosis of chest pain: a report of the American College of Cardiology/American Heart Association Joint Committee on Clinical Practice Guidelines. Circulation 144:e368–e454 34709879 10.1161/CIR.0000000000001029

[3] Kontos MC, de Lemos JA, Deitelzweig SB et al (2022) 2022 ACC expert consensus decision pathway on the evaluation and disposition of acute chest pain in the emergency department: a report of the American College of Cardiology Solution Set Oversight Committee. J Am Coll Cardiol 80:1925–1960 36241466 10.1016/j.jacc.2022.08.750 PMC10691881

[4] Monica MP, Merkely B, Szilveszter B, Drobni ZD, Maurovich-Horvat P (2020) Computed tomographic angiography for risk stratification in patients with acute chest pain—the triple rule-out concept in the emergency department. Curr Med Imaging Rev 16:98–110 32003310 10.2174/1573405614666180604095120

[5] Araoz PA, Gadam S, Bhanushali AK et al (2025) Triple rule out CT in the emergency department: clinical risk and outcomes. Acad Radiol 32:1297–1305 39658473 10.1016/j.acra.2024.10.051

[6] Russo V, Sportoletti C, Scalas G et al (2021) The triple rule out CT in acute chest pain: a challenge for emergency radiologists? Emerg Radiol 28:735–742 33604768 10.1007/s10140-021-01911-8 PMC8280047

[7] Wang K, Wang X, Zheng S, Li C, Jin L, Li M (2022) Dedicated CCTA followed by high-pitch scanning versus TRO-CT for contrast media and radiation dose reduction: a retrospective study. Diagnostics (Basel) 12:2647 36359488 10.3390/diagnostics12112647 PMC9688948

[8] Smith-Bindman R, Chu PW, Azman Firdaus H et al (2025) Projected lifetime cancer risks from current computed tomography imaging. JAMA Intern Med 185:710–719 40227719 10.1001/jamainternmed.2025.0505 PMC11997853

[9] Jakobs TF, Becker CR, Ohnesorge B et al (2002) Multislice helical CT of the heart with retrospective ECG gating: reduction of radiation exposure by ECG-controlled tube current modulation. Eur Radiol 12:1081–1086 11976849 10.1007/s00330-001-1278-x

[10] Leschka S, Scheffel H, Desbiolles L et al (2007) Image quality and reconstruction intervals of dual-source CT coronary angiography: recommendations for ECG-pulsing windowing. Invest Radiol 42:543–549 17620936 10.1097/RLI.0b013e31803b93cf

[11] Caruso D, De Santis D, Tremamunno G et al (2025) Deep learning reconstruction algorithm and high-concentration contrast medium: feasibility of a double-low protocol in coronary computed tomography angiography. Eur Radiol 35:2213–2221 39299952 10.1007/s00330-024-11059-x PMC11913928

[12] Sommer WH, Schenzle JC, Becker CR et al (2010) Saving dose in triple-rule-out computed tomography examination using a high-pitch dual spiral technique. Invest Radiol 45:64–71 20027121 10.1097/RLI.0b013e3181c15842

[13] Finck T, Klambauer K, Hendrich E, Will A, Martinoff S, Hadamitzky M (2021) Radiation dose and image quality of a high-pitch prospective spiral first approach in coronary computed tomography angiography (CCTA). J Cardiovasc Dev Dis 8:119 34677188 10.3390/jcdd8100119 PMC8539421

[14] Schönfeld T, Seitz P, Krieghoff C et al (2024) High-pitch CT pulmonary angiography (CTPA) with ultra-low contrast medium volume for the detection of pulmonary embolism: a comparison with standard CTPA. Eur Radiol 34:1921–1931 37656178 10.1007/s00330-023-10101-8 PMC10873234

[15] Fogante M, Esposto Pirani P, Cela F et al (2023) Ultra-low radiation dose and contrast volume CT protocol and TAVI-CT score for TAVI planning and outcome. Br J Radiol 96:20221026 37183830 10.1259/bjr.20221026 PMC10392642

[16] Hachulla AL, Ronot M, Noble S, Becker CD, Montet X, Vallee JP (2016) ECG-triggered high-pitch CT for simultaneous assessment of the aorta and coronary arteries. J Cardiovasc Comput Tomogr 10:407–413 27451049 10.1016/j.jcct.2016.07.010

[17] Apfaltrer P, Hanna EL, Schoepf UJ et al (2012) Radiation dose and image quality at high-pitch CT angiography of the aorta: intraindividual and interindividual comparisons with conventional CT angiography. AJR Am J Roentgenol 199:1402–1409 23169737 10.2214/AJR.12.8652

[18] Bamberg F, Marcus R, Sommer W et al (2012) Diagnostic image quality of a comprehensive high-pitch dual-spiral cardiothoracic CT protocol in patients with undifferentiated acute chest pain. Eur J Radiol 81:3697–3702 21196093 10.1016/j.ejrad.2010.11.032

[19] Leipsic J, Abbara S, Achenbach S et al (2014) SCCT guidelines for the interpretation and reporting of coronary CT angiography: a report of the Society of Cardiovascular Computed Tomography Guidelines Committee. J Cardiovasc Comput Tomogr 8:342–358 25301040 10.1016/j.jcct.2014.07.003

[20] Halpern EJ, Levin DC, Zhang S, Takakuwa KM (2009) Comparison of image quality and arterial enhancement with a dedicated coronary CTA protocol versus a triple rule-out coronary CTA protocol. Acad Radiol 16:1039–1048 19523852 10.1016/j.acra.2009.03.013

[21] Klok FA, Barco S, Bertoletti L et al (2025) Optimal approach to performing and reporting computed tomography angiography for suspected acute pulmonary embolism: a clinical consensus statement. Radiology 316:e243833 10.1148/radiol.243833 40459417

[22] Abbara S, Blanke P, Maroules CD et al (2016) SCCT guidelines for the performance and acquisition of coronary computed tomographic angiography: a report of the Society of Cardiovascular Computed Tomography Guidelines Committee: endorsed by the North American Society for Cardiovascular Imaging (NASCI). J Cardiovasc Comput Tomogr 10:435–449 27780758 10.1016/j.jcct.2016.10.002

[23] Holmquist F, Hansson K, Pasquariello F, Bjork J, Nyman U (2009) Minimizing contrast medium doses to diagnose pulmonary embolism with 80-kVp multidetector computed tomography in azotemic patients. Acta Radiol 50:181–193 19169917 10.1080/02841850802657269

[24] Schicchi N, Fogante M, Pirani PE et al (2020) Third generation dual source CT with ultra-high pitch protocol for TAVI planning and coronary tree assessment: feasibility, image quality and diagnostic performance. Eur J Radiol 122:108749 31759224 10.1016/j.ejrad.2019.108749

[25] Takx RAP, Krissak R, Fink C et al (2017) Low-tube-voltage selection for triple-rule-out CTA: relation to patient size. Eur Radiol 27:2292–2297 27686566 10.1007/s00330-016-4607-9 PMC5408040

[26] Chen Y, Wang Q, Li J, Jia Y, Yang Q, He T (2018) Triple-rule-out CT angiography using two axial scans with 16 cm wide-detector for radiation dose reduction. Eur Radiol 28:4654–4661 29789908 10.1007/s00330-018-5426-y

[27] Schockel L, Jost G, Seidensticker P, Lengsfeld P, Palkowitsch P, Pietsch H (2020) Developments in X-ray contrast media and the potential impact on computed tomography. Invest Radiol 55:592–597 32701620 10.1097/RLI.0000000000000696

[28] McDonald JS, McDonald RJ (2024) Risk of acute kidney injury following IV iodinated contrast media exposure: 2023 update, from the AJR special series on contrast media. AJR Am J Roentgenol 223:e2330037 37791729 10.2214/AJR.23.30037

[29] Rajiah PS, Dunning CAS, Rajendran K et al (2023) High-pitch multienergy coronary CT angiography in dual-source photon-counting detector CT scanner at low iodinated contrast dose. Invest Radiol 58:681–690 36822655 10.1097/RLI.0000000000000961 PMC10591289

[30] Araki S, Nakamura S, Takafuji M, Ichikawa Y, Sakuma H, Kitagawa K (2024) Ultra-low-dose coronary computed tomography angiography using photon-counting detector computed tomography. Eur Heart J Imaging Methods Pract 2:qyae125 39659699 10.1093/ehjimp/qyae125 PMC11631184

[31] Pannenbecker P, Heidenreich JF, Huflage H et al (2024) The best of both worlds: ultra-high-pitch pulmonary angiography with free-breathing technique by means of photon-counting detector CT for diagnosis of acute pulmonary embolism. Acad Radiol 31:4928–4937 10.1016/j.acra.2024.06.028 38969575

[32] Euler A, Higashigaito K, Mergen V et al (2022) High-pitch photon-counting detector computed tomography angiography of the aorta: intraindividual comparison to energy-integrating detector computed tomography at equal radiation dose. Invest Radiol 57:115–121 34352805 10.1097/RLI.0000000000000816

