# Supplementary material for: High-pitch free-breathing triple rule-out CT angiography for acute chest pain: a prospective sequential two-cohort comparison of radiation dose and diagnostic image quality
Source: Insights Imaging. 2026 Sep 4;17:221. doi: 10.1186/s13244-026-02390-6 (PMC13545161; doi:10.1186/s13244-026-02390-6)
Supplement: Supplementary file 1 [file 13244_2026_2390_MOESM1_ESM.pdf]

High-Pitch Free-Breathing Triple Rule-Out CT Angiography for Acute Chest Pain: A Prospective Sequential Two-Cohort Comparison of Radiation Dose and Diagnostic Image Quality

ELECTRONIC SUPPLEMENTARY MATERIAL

Insights Imaging (2026) Yu J, Peng Z, Yu X et al.

Table S1 Comparison of SNR and CNR between High-Pitch Triple Rule-Out CT Angiography (TRO-CTA) and Standard TRO-CTA

Criteria	Group A	Group B	*p* value
SNR			
CA-LM	13.86±2.71	36.32±11.92	< 0.001
CA-LAD	15.48±14.02	17.31±13.15	0.496
CA-LCX	20.72±34.57	19.28±15.37	0.784
CA-RCA	20.51±20.41	23.88±11.84	0.305
PA-MA	15.93±3.51	40.58±13.75	< 0.001
PA-LPA	14.68±4.41	34.17±11.44	< 0.001
PA-RPA	12.30±3.10	27.15±10.74	< 0.001
PA-LULA	19.93±9.40	38.36±20.53	< 0.001
PA-LLLA	16.29±6.11	40.65±16.33	< 0.001
PA-RULA	17.42±6.92	35.38±19.60	< 0.001
PA-RMLA	18.66±7.07	38.98±18.49	< 0.001
PA-RLLA	17.89±6.05	42.86±14.70	< 0.001
TA-AA	15.74±3.49	40.00±15.55	< 0.001
TA-DA	13.88±2.89	39.36±13.48	< 0.001
CNR			
CA-LM	32.87±12.02	67.64±22.47	< 0.001
CA-LAD	27.19±9.68	55.70±17.85	< 0.001
CA-LCX	27.43±8.02	54.88±17.59	< 0.001

CA-RCA	29.84±10.43	57.99±18.55	< 0.001
PA-MA	14.94±6.54	31.11±13.47	< 0.001
PA-LPA	13.80±5.96	29.34±12.70	< 0.001
PA-RPA	14.22±6.26	30.10±13.15	< 0.001
PA-LULA	13.70±6.22	28.51±12.67	< 0.001
PA-LLLA	13.36±6.31	27.92±12.37	< 0.001
PA-RULA	14.04±6.63	30.24±13.21	< 0.001
PA-RMLA	13.39±6.54	29.50±13.75	< 0.001
PA-RLLA	13.55±6.55	28.76±12.80	< 0.001
TA-AA	14.81±4.29	33.77±14.24	< 0.001
TA-DA	13.37±3.88	31.48±13.59	< 0.001

CA-LM, left main coronary artery; *CA-LAD*, left anterior descending artery; *CA-LCX*, left circumflex artery; *CA-RCA*, right coronary artery; *PA-MA*, main pulmonary artery; *PA-LPA*, left main pulmonary artery; *PA-RPA*, right main pulmonary artery; *PA-LULA*, left upper lobar artery; *PA-LLLA*, left lower lobar artery; *PA-RULA*, right upper lobar artery; *PA-RLLA*, right lower lobar artery; *PA-RMLA*, right middle lobar artery; *TA-AA*, ascending aorta; *TA-DA*, descending aorta.

Table S2 Clinical Diagnoses Identified by TRO-CTA in Both Groups

Diagnosis	Group A (n = 52)	Group B (n = 52)	p value
Coronary artery disease			0.752 [a]
Obstructive (severe stenosis or prior stenting)	4 (7.7)	6 (11.5)	
Moderate stenosis (50–70%)	7 (13.5)	7 (13.5)	
Mild/incidental [b]	29 (55.8)	24 (46.2)	
Unremarkable	12 (23.1)	15 (28.8)	
Acute aortic syndrome	4 (7.7)	4 (7.7)	1.000 [c]
Aortic intramural hematoma	0 (0)	1 (1.9)	
Penetrating aortic ulcer	4 (7.7)	3 (5.8)	
Pulmonary embolism	1 (1.9)	4 (7.7)	0.363 [c]
Any TRO acute finding [d]	8 (15.4)	12 (23.1)	0.455 [a]

※Data are presented as n (%)

[a] Chi-square test

[b] Mild/incidental findings include mild stenosis, myocardial bridging, and non-obstructive plaque

[c] Fisher's exact test

[d] Any TRO acute finding includes obstructive coronary artery disease, pulmonary embolism, or acute aortic syndrome. Three patients had ≥2 concurrent acute findings: one in Group A (obstructive CAD + PAU), and two in Group B (obstructive CAD + PAU; obstructive CAD + PE).

TRO, triple-rule-out; *CAD*, coronary artery disease; *PE*, pulmonary embolism; *AAS*, acute aortic syndrome; *PAU*, penetrating aortic ulcer
